# Fecal Microbiota Transfer from Young Mice Reverts Vascular Aging Hallmarks and Metabolic Impairments in Aged Mice

**DOI:** 10.14336/AD.2024.0384

**Published:** 2025-06-10

**Authors:** Chak Kwong Cheng, Jun Gao, Lijing Kang, Yu Huang

**Affiliations:** ^1^Department of Biomedical Sciences, City University of Hong Kong, Tat Chee Avenue, Hong Kong SAR, China.; ^2^Affiliated Qingyuan Hospital, The Sixth Clinical Medical School, Guangzhou Medical University, Qingyuan People’s Hospital, Guangdong, China.

**Keywords:** Aging, endothelial function, fecal microbiota transfer, intestine, sirtuin 1, telomere

## Abstract

As a major risk factor for cardiometabolic diseases, aging refers to a gradual decline in physiological function, characterized with 12 conspicuous hallmarks, like telomere attrition, chronic inflammation, and dysbiosis. Common vascular aging hallmarks include endothelial dysfunction, telomere dysfunction, and vascular inflammation. In this study, we sought to test the hypothesis that young-derived gut microbiota retards vascular aging hallmarks and metabolic impairments in aged hosts. We also aimed to study the therapeutic efficacy of young microbiota in hosts of different ages. Fecal microbiota transplantation (FMT) from young to aged or middle-aged C57BL/6 mice was conducted for 6 consecutive weeks after antibiotic pretreatment. Endothelium-dependent relaxations (EDRs) in mouse arteries were determined by wire myography. Inflammation and AMPK/SIRT1 signaling in mouse aortas and intestines were studied by biochemical assays. The telomere function of aortas and intestines, in terms of telomerase reverse transcriptase expression, telomerase activity, and relative telomere length, were also studied. FMT significantly reverted vascular dysfunction and metabolic impairments in middle-aged mice than in aged mice. Besides, FMT significantly reverted inflammation and telomere dysfunction in aortas and intestines of middle-aged mice. Improved intestinal barrier function and activated AMPK/SIRT1 signaling potentially underlie benefits of FMT. The findings imply gut-vascular connection as potential target against age-associated cardiometabolic disorders, highlight crosstalk among aging hallmarks, and suggest a critical timepoint for efficacy of anti-aging interventions.

## INTRODUCTION

Aging refers to a gradual decline of physiological integrity, along with augmented risks of diseases and mortality [1]. Until 2023, López-Otín *et al.* characterized aging with 12 critical hallmarks, including genomic instability, telomere attrition, deregulated nutrient-sensing, loss of proteostasis, mitochondrial dysfunction, disabled macroautophagy, epigenetic alterations, chronic inflammation, altered intercellular communication, cellular senescence, stem cell exhaustion, and dysbiosis [2]. A growing number of studies are exploring the comprehensive network among these critical hallmarks during the aging process and the development of age-associated diseases.

Vascular aging greatly increases the risk of cardiovascular morbidity. Common hallmarks of vascular aging include endothelial dysfunction, chronic inflammation, enhanced oxidative stress, increased vascular stiffness, and telomere dysfunction [3]. As a newly recognized hallmark of aging, *‘dysbiosis’* significantly alters host homeostasis, contributory to altered inflammatory and immune responses [2]. Additional to cardiovascular diseases, age-associated dysbiosis elevates the risks of numerous diseases, like cancers, diabetes, and Parkinson’s disease [4]. However, the comprehensive mechanisms of how dysbiosis interacts with other aging hallmarks to aggravate the progression of age-associated diseases remain elusive.

**Figure 1.**
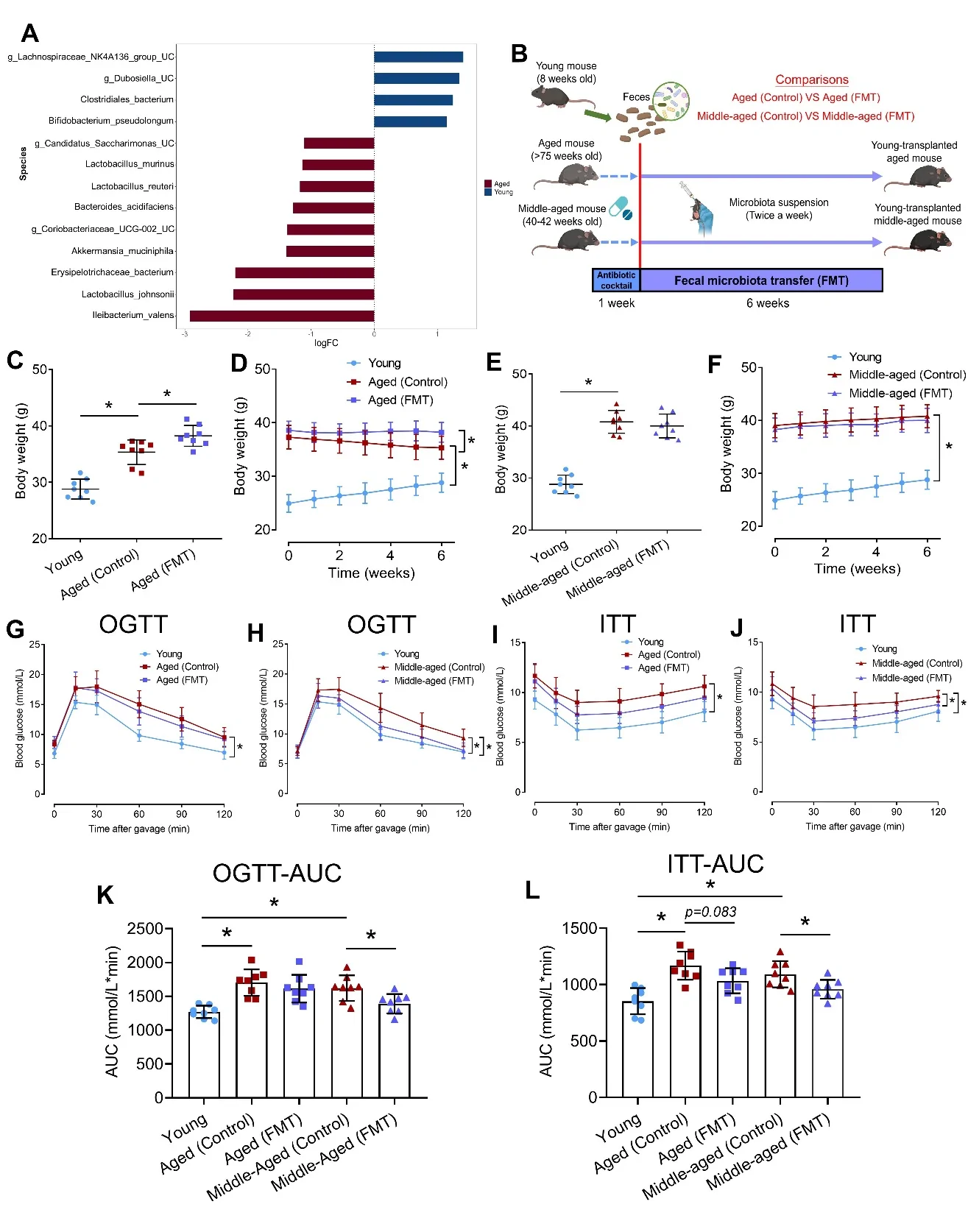
**Metabolic profiles of recipient mice upon young-to-aged FMT**. (**A**) Differential abundance analysis on the mean difference in centered log ratio for enriched species in young and aged mice (n=8 per group). (**B**) Schematic overview on FMT protocol from young donor mice to aged recipient mice. (**C**) Body weights of young donor mice (Young), aged control mice (Aged (Control)) and young-transplanted aged mice (Aged (FMT)) after 6-week FMT protocol (n=8 per group). (**D**) Body weight changes of mice in (**C**) during the 6-week FMT (n=8 per group). (**E**) Body weights of young, middle-aged control mice (Middle-aged (Control)) and young-transplanted middle-aged mice (Middle-aged (FMT)) after 6-week FMT protocol (n=8 per group). (**F**) Body weight changes of mice in (**E**) during the 6-week FMT (n=8 per group). OGTT of (**G**) young and aged mice, and (**H**) young and middle-aged mice at week 6 of FMT (n=8 per group). ITT of (**I**) young and aged mice, and (**J**) young and middle-aged mice at week 6 of FMT (n=8 per group). AUC analysis on (**K**) OGTT, and (**L**) ITT of glucose over time of young, aged, and middle-aged mice at week 6 of FMT (n=8 per group). The same body weight datasets for Young group in (**C-F**). The same curves on OGTT results for Young group in (**G**) and (**H**). The same curves on ITT for Young group in (**I**) and (**J**). Data are mean ± SD. *p<0.05 (D'Agostino-Pearson normality test, followed by unpaired t-test and nonparametric Mann-Whitney test). AUC, area under curve; FMT, fecal microbiota transfer; ITT, insulin tolerance test; OGTT, oral glucose tolerance test.

The close proximity between intestine and blood circulation implies susceptibility of vasculature towards age-associated dysbiosis, hinting the regulatory role of gut-vascular connection on cardiovascular homeostasis during aging [5]. For instance, a previous study has shown that depletion of gut microbiota by antibiotics in aged mice alleviates vascular dysfunction and vascular oxidative stress [6], implying exacerbation of vascular aging by dysbiosis. Our preliminary findings also showed that fecal microbiota transfer (FMT) from aged mice induced endothelial dysfunction and vascular inflammation in young mice (unpublished). However, whether FMT from young mice alleviates vascular aging hallmarks in aged mice remains unclear. Besides, the pace of aging becomes substantially higher after certain critical timepoints in life [7], hinting that the efficacy to retard or even revert aging might markedly drop after certain timepoints. We therefore hypothesize that young microbiota might revert vascular aging hallmarks in aged host, and such reversal effect is age-dependent, meaning that the reversal efficacy might greatly reduce in advanced age.

Therefore, in the present study, we conducted young-to-aged FMT to (i) study the reversal effects of young microbiota on vascular function and metabolism of aged hosts at different ages, and (ii) provide rapid mechanistic insights into how young microbiota might benefit the vasculature and intestine. Our findings shall provide novel insights that the gut-vascular connection can potentially be an intervention target, thus opening up new opportunities for development of alternative therapeutic strategies against age-associated cardiometabolic diseases. Our findings also imply that the timing of anti-aging interventions is critical for optimizing the therapeutic window.

## MATERIALS AND METHODS

### Ethical statements

All experiments in this study were conducted in compliance with the ARRIVE guidelines, and the Guide for the Care and Use of Laboratory Animals issued by the National Institutes of Health. All animal experiments were performed in compliance with the procedures and ethical polices established by the Animal Research Ethics Sub-Committee, City University of Hong Kong (approval No. AN-STA-00000132).

Materials and Methods are available in the online-only Supplementary Material.

## RESULTS AND DISCUSSION

### Young-to-aged FMT age-dependently improved metabolic profiles

We first studied the gut microbial profiles in aged and young mice before the FMT protocol. 16S rRNA sequencing revealed different microbial profiles in aged and young mice, where certain bacterial species, such as *Lactobacillus murinus*, *Lactobacillus reuteri*, *Bacteroides acidifaciens*, *Akkermansia muciniphila*, *Erysipelo-trichaceae bacterium*, *Lactobacillus johnsonii*, and *Ileibacterium valens*, exhibited significant enrichment in aged versus young mice (Fig. 1A). Meanwhile, *Bifidobacterium pseudolongum* was significantly enriched in young versus aged mice, consistent with human findings that the abundance of *Bifidobacterium* species declines in aged population [8]. We then conducted FMT from young mice (8 weeks old) to middle-aged (40-42 weeks old) and aged mice (>75 weeks old) to investigate the effects of young microbiota on aged (Aged (Control) VS Aged (FMT)) and middle-aged hosts (Middle-aged (Control) VS Middle-aged (FMT)) (Fig. 1B). In other words, comparisons were particularly made between FMT and Control groups of aged and middle-aged mice respectively to understand the cardiometabolic effects of young microbiota (Fig. 1B). During advanced aging, gradual weight loss is common in humans and rodents [9]. FMT slightly retarded weight loss in aged but not in middle-aged mice (Fig. 1C-F; Supplementary Fig. 1A-B), without altering the amounts of food intake (Supplementary Fig. 1C-D), hinting that microbiota remodeling mainly accounted for the weight change. The slightly higher weights of certain organs and adipose tissues contributed to the weight-loss retardation in young-transplanted aged mice (Supplementary Fig. 2), suggesting a systemic effect of FMT. After 3 weeks of FMT, insulin resistance was slightly reduced in middle-aged but not in aged mice, when compared to corresponding control mice (Supplementary Fig. 3). After the 6-week FMT, while insulin resistance was slightly reduced in aged mice (Aged (FMT) VS Aged (Control)), both glucose tolerance and insulin resistance were significantly improved in middle-aged mice (Middle-aged (FMT) VS Middle-aged (Control)) (Fig. 1G-L). Moreover, FMT significantly raised high-density lipoprotein (HDL) levels but lowered low-density lipoprotein (LDL) levels in sera of middle-aged mice (Supplementary Fig. 4). These findings suggested differentially altered metabolic profiles in aged and middle-aged mice upon receiving young microbiota.

**Figure 2.**
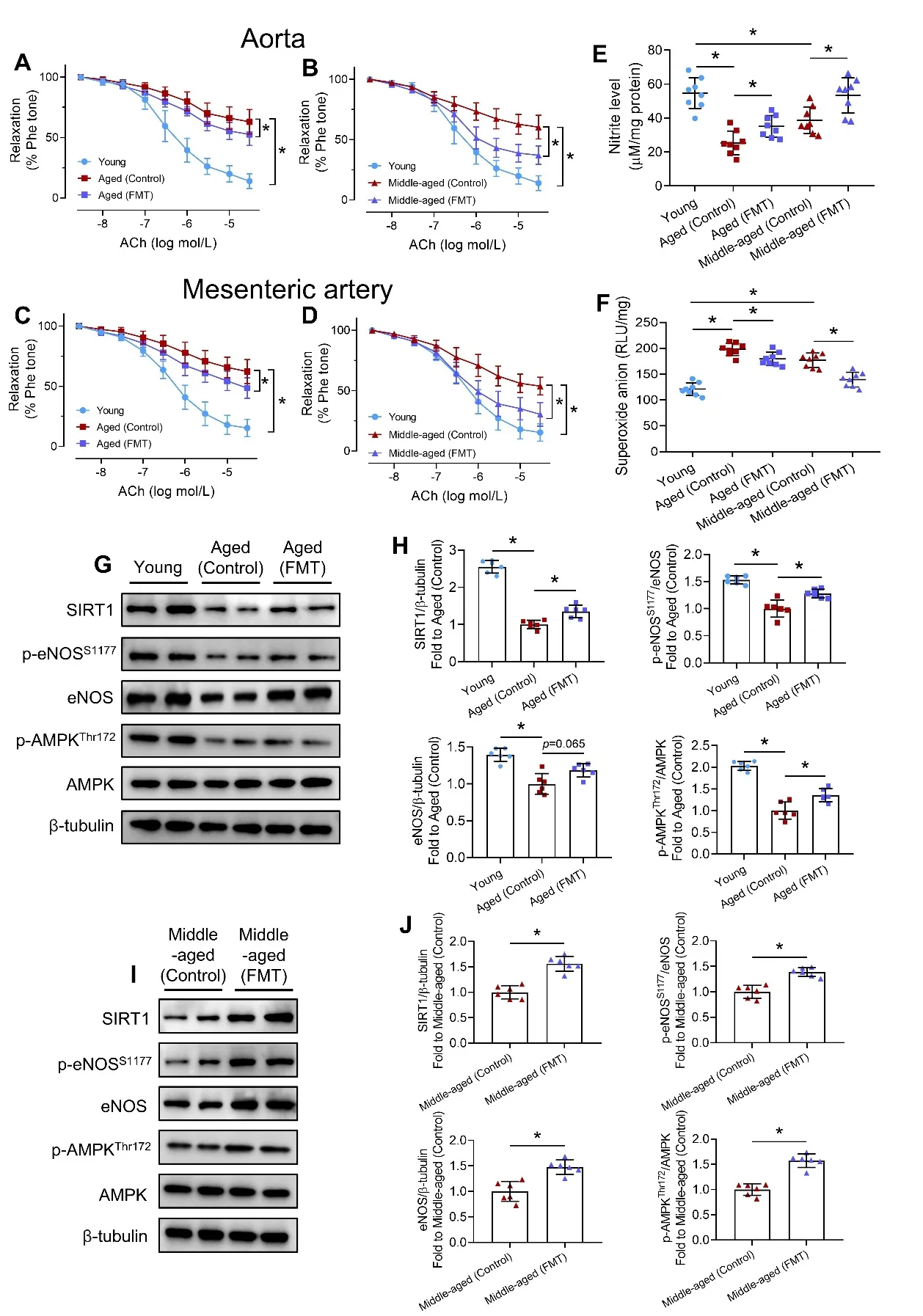
**Vascular function of recipient mice upon young-to-aged FMT**. Summary statistics of wire myography on EDRs in aortas of (**A**) young and aged mice, and (**B**) young and middle-aged mice (n=8 per group). Summary statistics of wire myography on EDRs in mesenteric arteries of (**C**) young and aged mice, and (**D**) young and middle-aged mice (n=8 per group). (**E**) Nitrite levels in aortas of different mouse groups (n=8 per group). (**F**) Lucigenin-enhanced chemiluminescence on aortic ROS levels of different mouse groups (n=8 per group). Representative Western blots on expression of AMPK, p-AMPK at Thr172, eNOS, p-eNOS at Ser1177 and SIRT1 in aortas of (**G**) young and aged mice, and (**H**) corresponding quantification (n=6 per group). Representative Western blots on expression of AMPK, p-AMPK at Thr172, eNOS, p-eNOS at Ser1177 and SIRT1 in aortas of (**I**) middle-aged mice, and (**J**) corresponding quantification (n=6 per group). The same curves on EDRs of aortas for Young group in (**A**) and (**B**). The same curves on EDRs of mesenteric arteries for Young group in (**C**) and (**D**). Data are mean ± SD. *p<0.05 (D'Agostino-Pearson normality test, followed by unpaired t-test and nonparametric Mann-Whitney test). ACh, acetylcholine; EDR, endothelium-dependent relaxation; FMT, fecal microbiota transfer; Phe, phenylephrine.

### Young-to-aged FMT age-dependently improved vascular function

Since undesirable metabolic changes, especially hyperglycemia and dyslipidemia, are crucial factors causing endothelial damage [10], we postulated that endothelial function of recipient mice might be differentially altered following the observed patterns in blood glucose and lipid profile measurements. Importantly, young-to-aged FMT more remarkably improved endothelium-dependent relaxations (EDRs) in aortas of middle-aged mice (Fig. 2A-B; Supplementary Fig. 5), without altering sodium nitroprusside (SNP)-induced endothelium-independent relaxations (Supplementary Fig. 6), implying that the improved vascular function was mainly due to endothelium-derived NO. Young-to-aged FMT might have altered the metabolite pool in circulation (e.g. glucose, lipids and other factors) to influence endothelial function. Besides, FMT significantly improved EDRs in mesenteric arteries of middle-aged mice (Fig. 2C-D; Supplementary Fig. 7). Relaxations of mesenteric arteries depend on both endothelial nitric oxide synthase (eNOS)-derived NO and endothelium-derived hyperpolarizing factor (EDHF). NG-nitro-L-arginine methyl ester (L-NAME) was used to eliminate eNOS-derived NO production for evaluating the role of EDHF in mesenteric arteries. The unaltered EDHF-dependent vasodilation in mesenteric arteries indicated that the improved relaxations were mainly due to increase NO bioavailability (Supplementary Fig. 8)[11]. Consistently, nitrite assay revealed higher nitrite levels, indicating elevated NO production in aortas of young-transplanted mice (Fig. 2E). Additionally, lucigenin-enhanced chemiluminescence showed lower ROS levels in aortas of middle-aged mice undergoing FMT (Fig. 2F), suggesting less NO quenching for higher NO bioavailability. In other words, FMT more remarkably suppresses vascular oxidative stress in middle-aged mice, implying the lowering of harmful factors that are potentially pro-oxidant in circulation.

During aging, vascular function-related signaling becomes dysregulated [12]. eNOS and AMP-activated protein kinase (AMPK) phosphorylation, and sirtuin 1 (SIRT1) expression were found gradually downregulated in mouse aortas during aging (Supplementary Fig. 9). Notably, AMPK and SIRT1 signaling partnership is involved in longevity regulation in various cells and tissues [13]. Importantly, FMT more significantly upregulated phospho-eNOS (S1177) and total eNOS expression in aortas of middle-aged mice compared to aged mice (Fig 2G-J), implying more eNOS/NO production in endothelium upon FMT. As upstream regulators of eNOS, aortic expression of phospho-AMPK (Thr172) and SIRT1 were found upregulated upon FMT (Fig. 2G-J), suggesting activated AMPK/eNOS, SIRT1/eNOS, and AMPK/SIRT1 signaling in enhancing endothelial function, and potentially retarding vascular aging [13]. These findings implied the age-dependent vascular benefits of young microbiota. Receiving young microbiota at younger age might be of higher therapeutic efficacy in vasculature.

### Young-to-aged FMT age-dependently retarded vascular inflammation and telomere dysfunction

**Figure 3.**
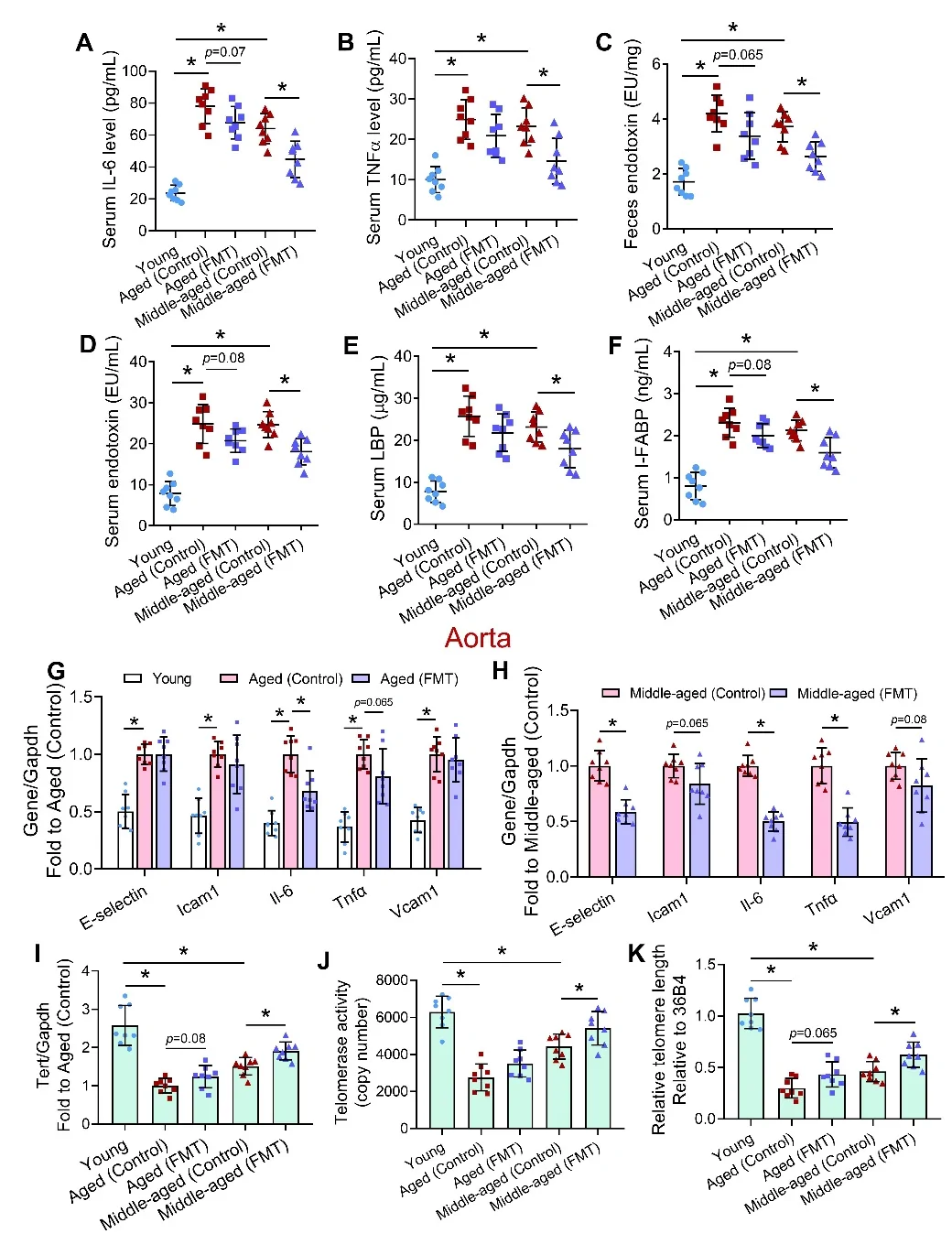
**Effects of young-to-aged FMT on systemic and vascular inflammation, and vascular telomere function**. ELISA on circulating inflammatory marker (**A**) IL-6, and (**B**) TNFα of different mouse groups (n=8 per group). Endotoxin levels in (**C**) feces and (**D**) serum of different mouse groups (n=8 per group). ELISA on serum levels of (**E**) LBP and (**F**) I-FABP of different mouse groups (n=8 per group). RT-PCR on mRNA levels of pro-inflammatory genes in aortas of (**G**) young and aged mice, and (**H**) middle-aged mice (n=8 per group). (**I**) Tert mRNA level, (**J**) telomerase activities and (**K**) relative telomere length in aortas of different mouse groups (n=8 per group). Data are mean ± SD. *p<0.05 (D'Agostino-Pearson normality test, followed by unpaired t-test and nonparametric Mann-Whitney test). FMT, fecal microbiota transfer; I-FABP, intestinal fatty acid binding protein; LBP, LPS-binding protein; Tert, telomerase reverse transcriptase.

Chronic inflammation is a hallmark of vascular aging and a driver of endothelial dysfunction, where the tight partnership between inflammation and oxidative stress further exacerbates age-associated vascular damage [14]. Since microbiota remodeling causes inflammatory alterations in hosts and age-associated dysbiosis promotes systemic inflammation [15], we wondered whether receiving young microbiota could retard these harmful events in aged hosts. ELISA results showed more remarkably downregulated serum levels of pro-inflammatory cytokines (interleukin (IL)-6 and tumor necrosis factor α (TNFα)) in middle-aged mice upon FMT (Fig. 3A-B), implying the suppressive effect of FMT on systemic inflammation. Notably, aging is associated with increased intestinal permeability, causing endotoxemia [16]. Fecal and serum endotoxin levels, and serum LPS-binding protein (LBP) levels were more significantly lowered in FMT-receiving middle-aged mice (Fig. 3C-E). Besides, serum level of intestinal fatty-acid binding protein (I-FABP), the surrogate biomarker of increased intestinal permeability [17], were more significantly lowered in FMT-receiving middle-aged mice (Fig. 3F). These findings suggested the anti-inflammatory effect of young-to-aged FMT through retarding ‘*leaky gut*’ and endotoxemia. Moreover, the reduced endotoxemia is believed to alleviate endothelial damage, vascular oxidative stress, and vascular inflammation [18], and improve host glucose homeostasis [19]. Systemic inflammation can mediate local inflammation. Consistent to the suppressed systemic inflammation, FMT more remarkably suppressed expression of pro-inflammatory markers in aortas of middle-aged mice (Fig. 3G-H), elucidating mitigated vascular inflammation.

**Figure 4.**
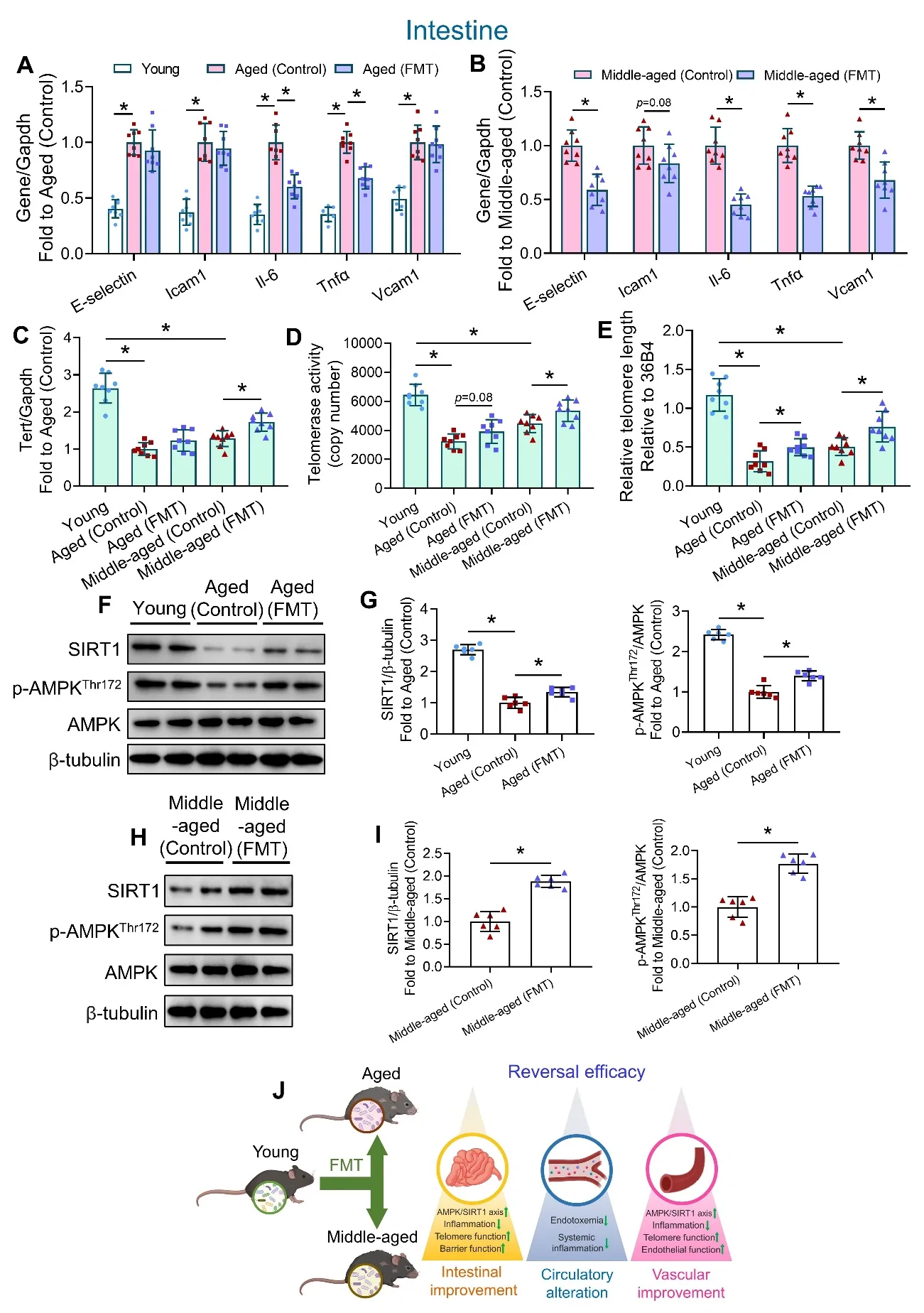
**Effects of young-to-aged FMT on inflammation, telomere function and AMPK/SIRT1 signaling in intestines**. RT-PCR on mRNA levels of pro-inflammatory genes in intestines of (**A**) young and aged mice, and (**B**) middle-aged mice (n=8 per group). (**C**) Tert mRNA level, (**D**) telomerase activities and (**E**) relative telomere length in intestines of different mouse groups (n=8 per group). Representative Western blots on expression of AMPK, p-AMPK at Thr172 and SIRT1 in intestines of (**F**) young and aged mice, and (**G**) corresponding quantification (n=6 per group). Representative Western blots on expression of AMPK, p-AMPK at Thr172 and SIRT1 in intestines of (**H**) middle-aged mice, and (**I**) corresponding quantification (n=6 per group). Data are mean ± SD. *p<0.05 (D'Agostino-Pearson normality test, followed by unpaired t-test and nonparametric Mann-Whitney test). (**J**) Schematic overview on the reversal effects of FMT on aged and middle-aged mice. FMT, fecal microbiota transfer; Tert, telomerase reverse transcriptase.

Telomere attrition is a key aging hallmark, where chronic inflammation and oxidative damage promote telomere dysfunction [20]. Besides, SIRT1 is a critical regulator for telomere maintenance [21], where its aortic expression was upregulated upon FMT (Fig. 2G-J). We therefore evaluated telomere function in aortas. Our results showed that FMT more remarkably upregulated telomerase reverse transcriptase (Tert; Fig. 3I), a catalytic telomerase subunit, enhanced telomerase activity (Fig. 3J), and retarded shortening of relative telomere length (Fig. 3K) in aortas of middle-aged mice. Importantly, endothelial telomere dysfunction was previously shown to aggravate vascular inflammation and oxidative stress to induce endothelial dysfunction [22], implying a potential positive loop between inflammation and telomere dysfunction in accelerating aging. However, the reversal effect of young-to-aged FMT on telomere function was lower in aged mice when compared to middle-aged mice (Fig. 3I-K). Further studies are required to identify threshold timepoint for therapeutic optimization or to improve microbiota regimen for positive effects even in older age.

### Young-to-aged FMT age-dependently modulated inflammation, telomere function, and AMPK/SIRT1 signaling in intestine

Compared to the vasculature, the intestine is at the closest proximity to gut microbiota and microbiota-derived factors. During aging, gradual downregulation of AMPK phosphorylation and SIRT1 expression was observed in mouse intestines (Supplementary Fig. 10). It is therefore reasonable to postulate that young-to-aged FMT might elicit similar beneficial effects on intestine. FMT more significantly attenuated the expression of pro-inflammatory genes (Fig. 4A-B), and telomere dysfunction in intestines of middle-aged mice (Fig. 4C-E). Furthermore, intestinal expression of phospho-AMPK (Thr172) and SIRT1 were found upregulated upon FMT (Fig. 4F-I), where the activated AMPK/SIRT1 axis potentially retards intestinal aging. A positive loop might exist between inflammation and telomere dysfunction in aging intestine, requiring further molecular validation. Meanwhile, suppressed inflammation and AMPK activation were confirmed to improve intestinal integrity [23], consistent with our findings on reduced intestinal permeability and endotoxemia upon FMT. A recent study suggested SIRT1 involvement in regulating intestinal barrier function [24], but further detailed mechanistic study is needed. The mechanistic link between SIRT1 and telomere function in intestine also needs further investigation. Previous studies suggested that intestinal AMPK activation is beneficial to intestinal barrier function [23], and host metabolism [25]. However, a recent study suggested that intestinal AMPK activation increases ceramide production promoting liver disease progression [26]. The comprehensive role of intestinal AMPK/SIRT1 signaling remains elusive, where extensive metabolite profiling shall facilitate the identification of more critical metabolites downstream to the activation of AMPK/SIRT1 signaling. Future studies shall identify drugs and interventions that could mimic the effect of young microbiota in modulating AMPK/SIRT1 signaling.

The extent of telomere length preservation was slightly higher in intestine (~30%) than in aortas (~20%), hinting differential sensitivity to beneficial factors upon microbiota remodeling and differential mechanisms of telomere regulation in different organs/tissues. We cannot exclude the possibility that transplantation of young microbiota might elicit beneficial effects on other organs/tissues, and that the proximity between intestine and organs might influence therapeutic effects of FMT. Altogether, the novel findings highlight a tight gut-vascular connection in conferring the benefits of young microbiota.

In summary, we demonstrated that gut microbiota remodeling, achieved by young-to-aged FMT, age-dependently elicited beneficial effects on host vasculature and intestine (Fig. 4J). FMT significantly retarded metabolic impairments, and alleviated hallmarks of vascular aging, including endothelial dysfunction, inflammation, and telomere dysfunction, in middle-aged mice. Additionally, FMT remarkably retarded inflammation and telomere dysfunction in intestines of middle-aged mice, along with improved intestinal barrier integrity. The activated AMPK/SIRT1 signaling potentially underlies the beneficial effects of FMT. Further extensive studies are needed to investigate the potential long-term beneficial and side effects of young-associated FMT, particularly in terms of safety and therapeutic efficacy. Future efforts are required to study whether young microbiota brings about similar cardiometabolic benefits, like telomere regulation, through gut-vascular connection in human individuals. Altogether, these findings deepen our understanding towards the comprehensive network underlying different hallmarks of aging and provide therapeutic insight that gut-vascular connection potentially represents an intervention target against age-associated cardiometabolic diseases.

## Supplementary Materials

The Supplementary data can be found online at: www.aginganddisease.org/EN/10.14336/AD.2024.0384.

## Data Availability

The data that support the findings of this study are either included in the Manuscript and Supplementary Materials or available from the corresponding author upon reasonable request.
