# Supplementary material for: Fecal Microbiota Transfer from Young Mice Reverts Vascular Aging Hallmarks and Metabolic Impairments in Aged Mice
Source: Aging Dis. 2024 Jun 10;16(3):1576–85. doi: 10.14336/AD.2024.0384 (PMC12096931; doi:10.14336/AD.2024.0384)
Supplement: Supplementary file 1 — The Supplementary data can be found online at: www.aginganddisease.org/EN/10.14336/AD.2024.0384. [file AD-16-3-1576-s.pdf]

SUPPLEMENTARY DATA

Fecal Microbiota Transfer from Young Mice Reverts Vascular Aging Hallmarks and Metabolic Impairments in Aged Mice

Chak Kwong Cheng, Jun Gao, Lijing Kang, Yu Huang

© 2024. Cheng CK et al. Published online at http://www.aginganddisease.org/EN/10.14336/AD.2024.0384

SUPPLEMENTARY DATA

PART I: MATERIALS AND METHODS

Study design

The current animal study consisted of controlled laboratory experiments which aimed to study the vascular and metabolic effects of FMT from young mice in aged mice, to provide mechanistic insights underlying the effects, and to compare the reversal efficacy of fecal microbiota transfer (FMT) in mice of different ages. Endothelial function, certain metabolic parameters, vascular inflammation, telomere function and protein expression of recipient aged mice were studied. Experimental blinding was applied to reduce the risk of bias in the present study whenever possible. The drugs used for the experiments were prepared by laboratory members who did not perform the experiments. Meanwhile, all animals were randomized before treatment. Sample size, where the number was kept unaltered throughout the study, was determined based on our previous studies, and sample size and power calculator. The treatment protocol and downstream data collection would be terminated, and the corresponding data of the animals would be excluded once unfavorable health conditions are observed in experimental animals during treatment protocol or unexpected tumorigenesis is identified. GraphPad Prism's outlier test was applied to identify the presence of outliers. The endpoints were selected based on previous studies.

Animal studies and ethical statements

All experiments in this study were conducted in compliance with the ARRIVE guidelines, and the Guide for the Care and Use of Laboratory Animals issued by the National Institutes of Health. All animal experiments were performed in compliance with the procedures and ethical polices established by the Animal Research Ethics Sub-Committee, City University of Hong Kong (approval No. AN-STA-00000132). Specific pathogen-free (SPF) male C57BL/6 mice of different ages (young: 8 weeks old (n = 8); middle-aged: 40-42 weeks old (n = 16); aged: >75 weeks old (n = 16) were provided by Laboratory Animal Research Unit, City University of Hong Kong. All mice were housed in individually well-ventilated caging systems at constant temperature (23 ± 1°C) and humidity (55% ± 5%) in an SPF animal facility, under a 12h light: 12h dark cycle. The mice were allowed free access to laboratory food pellets and water. All mice were randomized before experiments.

Fecal microbiota transfer (FMT)

Before FMT protocol, aged and middle-aged control and recipient C57BL/6 mice were treated with an antibiotic cocktail in drinking water, containing 1 g/L ampicillin, 0.5 g/L neomycin, 1 g/L metronidazole and 0.5 g/L vancomycin, for 1 week. Starting from the first day after antibiotic administration, aged and middle-aged recipient mice were given via oral gavage 150 μL of microbiota suspension from young donors C57BL/6 mice twice a week for 6 consecutive weeks, which are equivalent to 4.667 human years (9 mouse days~1 human year; Fig. 1B). Meanwhile, aged, and middle-aged control mice were orally gavaged with 150 μL of microbiota suspension from age-matched aged or middle-aged donor C57BL/6 mice twice a week for 6 weeks. To prepare microbiota suspension, fresh fecal pellets from 10-12 young donor mice were pooled together. The pellets were weighed and resuspended by vortex in sterile PBS (1 mL/300 mg feces) for 1 min. The insolubilized material was pelleted by centrifugation at 500 xg for 5 min, and the supernatant was used for FMT. Additionally, the cages of aged and middle-aged recipient mice were replenished with fresh fecal pellets and dirty bedding from young donor mice once a week. Meanwhile, the cages of aged and middle-aged control mice were also replenished with fresh fecal pellets and dirty bedding from age-matched aged and middle-aged mice once a week. During the 6-week FMT, body weights and the amount of food intake of mice were recorded weekly. Blood glucose levels were measured at 3rd and 6th weeks of FMT. The weights of organs (including heart, liver, lung, spleen, kidney and gastrocnemius) and weights of adipose tissues (including ingSAT, pgVAT and BAT) were measured after mouse scarification by CO_2 suffocation after the 6-week FMT.

Fecal DNA collection

Fecal pellets from young and aged mice were collected and stored at -80ºC for later processing. Bacterial DNA was extracted from fecal pellets by using the NucleoSpin® DNA Stool kit (Macherey-Nagel, Düren, Germany). In brief, 180-200 mg of fecal pellets were homogenized in bead beating tubes by vortex for 10 min at maximal speed. Total genomic DNA was captured by the silica membrane and impurities were subsequently removed by NucleoSpin® Inhibitor Removal Column. Purified DNA was then eluted by 80 μL Elution Buffer and DNA concentrations were determined by NanoDrop at 260 nm.

16S rRNA sequencing and data analysis

© 2024. Cheng CK et al. Published online at http://www.aginganddisease.org/EN/10.14336/AD.2024.0384

SUPPLEMENTARY DATA

DNA concentrations were measured by the Qubit® dsDNA HS Assay Kit. Preparation of next generation sequencing libraries and Illumina sequencing were performed by Genewiz, Inc. (South Plainfield, NJ). The sequencing library was constructed using a MetaVX Library Preparation Kit (Genewiz, Inc.). In brief, 20-30 ng of DNA was used to generate amplicons covering V3-V4 hypervariable regions of bacterial 16S rRNA gene, by using the primer pairs (forward: CCTACGGRRBGCASCAGKVRVGAAT; reverse (GGACTACVSGGGTATCTAAT). The PCR products were later purified by magnetic beads and the concentrations were measured by an Infinite 200 Pro microplate reader (Tecan, Männedorf, Switzerland). The fragment size was validated by 1.5% agarose gel electrophoresis. Next generation sequencing was then conducted on the Illumina Miseq/Novaseq Platform (Illumina, San Diego, USA) at Genewiz, Inc. Automated cluster generation and 250/300 paired-end sequencing with dual reads were performed according to the manufacturer's instructions.

The QIIME package was used for 16S rRNA data analysis. The forward and reverse reads were merged, assigned to samples based on barcode, and truncated by cutting off the primer and barcode sequence. Quality filtering on successfully merged reads was performed according to QIIME default settings. Sequences were classified into operational taxonomic units (OTUs) based on the similarity to annotated bacterial sequences using the clustering program VSEARCH (1.9.6; 97% sequence similarity cut-off). The Ribosomal Database Program (RDP) classifier was applied to assign taxonomic category to all OTUs (confidence threshold: 0.8).

Blood glucose measurement

Oral glucose tolerance test (OGTT) and insulin tolerance test (ITT) were conducted in mice after fasting (16h for OGTT and 2h for ITT). Following oral gavage of glucose (1.2 g/kg) or IP injection of insulin (1 unit/kg), the blood glucose levels were measured in venous blood from mouse tails at specified time points (0, 15, 30, 60, 90 and 120 min).

Wire myography

After mouse scarification by CO_2 suffocation, thoracic aortas and mesenteric arteries (2nd order) were dissected out for functional assay by wire myography. The adhering connective tissues were carefully removed in sterile PBS. The arteries were further dissected into ring segments (~2 mm in length) in ice-cold oxygenated Krebs solution containing (in mmol/L): 119 NaCl, 4.7 KCl, 25 $NaHCO_3$, 1.2 KH_2PO_4, 2.5 $CaCl_2$, 1 $MgCl_2$ and 11 D-glucose, and then individually mounted on a Multi Wire Myograph System (Danish Myo Technology, Hinnerup, Denmark) for isometric tension measurement. Each ring was initially stretched to an optimal baseline tension (aorta: 3 mN; mesenteric artery: 2 mN) and allowed to equilibrate for 1 hr at 37ºC with continuous oxygenation (95% O_2, 5% CO_2) in Krebs solution. The rings were pre-contracted by 60 mmol/L KCl and rinsed three times in Krebs solution. Then phenylephrine (Phe; 3 μmol/L; Sigma-Aldrich) was added to pre-contract the rings, and acetylcholine (Ach; 3 nmol/L to 10 μmol/L; Sigma-Aldrich) was cumulatively added to induce EDRs. Before EDRs, some mesenteric arteries were pre-incubated with the NOS inhibitor L-NAME (100 μmol/L; Sigma-Aldrich) for 30 min. Endothelium-independent relaxation was also assessed by cumulative additions of SNP (1 nmol/L to 10 μmol/L; Sigma-Aldrich). Changes in isometric tension were documented by the PowerLab LabChart 7.0 system (AD Instruments, Bella Vista, NSW, Australia).

Quantitative real-time PCR

Total RNA from aortic and intestinal tissues were extracted with TRIzol reagent (Invitrogen). cDNAs were synthesized by using the iScriptTM cDNA synthesis kit (Bio-Rad, Hercules, USA). Quantitative RT-PCR was performed by using SYBR Premix ExTaq (TaKaRa) in ABI ViiA7 system. Gapdh was used as endogenous control. Mouse primer pairs used in this study were listed in Supplementary Table 1.

Western blotting

Mouse aortic and intestinal tissues were homogenized in ice-cold RIPA lysis buffer (1X), supplemented with Complete Protease Inhibitor cocktail (Sigma-Aldrich) and phosSTOP phosphatase inhibitor (Roche, Basel, Switzerland). A BCA protein assay kit (Pierce Biotechnology, Rockford, USA) was used to determine protein concentrations. Equal amount of protein was loaded and separated by a 10% SDS-polyacrylamide gel and was then transferred to an Immobilon-P polyvinylidene difluoride membrane (Millipore Corp., Burlington, USA). The membranes were blocked in TBS (3% BSA) supplemented with 0.05% Tween-20 for 30 min, followed by overnight incubation at 4ºC with primary antibodies: anti-eNOS (1:1000; catalog number: 610297; BD Transduction Laboratory, San Diego, USA), anti-phospho-eNOS at Ser1177 (1:1000; catalog number: 9571S; Cell Signaling Technology, Danvers, USA), anti-AMPK (1:1000; catalog number: 2532; Cell Signaling Technology), anti-phospho-AMPK at Thr172 (1:1000; catalog number: 2535; Cell Signaling Technology), anti-SIRT1 (1:1000; catalog number: 9475; Cell Signaling Technology), and anti-β-tubulin (1:1000; catalog number: HC101;

Transgen Biotech, Beijing, China). Bound antibodies were incubated with horseradish peroxidase-conjugated anti-rabbit (1:3000; catalog number: 7074; Cell Signaling Technology) or anti-mouse secondary antibodies (1:3000; catalog number: 7076; Cell Signaling Technology) for 2h at room temperature. Protein bands were visualized by enhanced chemiluminescence (Cell Signaling Technology) by using ChemiDoc™ Imaging System (Bio-Rad).

Telomerase activity assay
Telomerase activity of aortic and intestinal tissues was measured by RT-PCR using the TRAPeze® RT Telomerase Detection Kit (Merck, Darmstadt, Germany). Briefly, telomerase was extracted by lysing tissues with CHAPS lysis buffer (200 μL/50 mg of tissues) by using mechanical homogenizer. Protein concentration was evaluated by Bradford method and normalized to 500 ng/μL. A master mix was prepared by mixing: Taq DNA Polymerase (5 units/μL; Thermo Scientific), 5x TRAPEZE® RT reaction mixes and nuclease-free water, and was then aliquoted to an RNase-free 96-well plate. Samples were randomized and assayed in duplicate. Standard curve on TSR8, positive and negative controls were examined accordingly. Telomerase activity value was extrapolated from the standard curve on serial dilutions of TSR8 control (1:10; 0.4–0.0004 attomoles).

Relative telomere length measurement

The relative telomere lengths in aortic and intestinal tissues were evaluated by RT-PCR. Genomic DNA was extracted by using genomic DNA purification kit (Thermo Scientific). RT-PCR was performed in ABI ViiA7 system, using specific primers for telomere and acidic ribosomal phosphoprotein (36B4) gene, a single copy conserved gene which served as internal control. The primer pairs for telomere and 36B4 gene were listed in Supplementary Table 1. The relative telomere length was calculated based on ΔCT values.

Lucigenin-enhanced chemiluminescence assay

Superoxide anion production in mouse aortas was determined by lucigenin-enhanced chemiluminescence assay. Freshly dissected mouse aortas were incubated in Krebs-HEPES solution containing (in mmol/L): 99 NaCl, 4.7 KCl, 25 $NaHCO_3$, 20 Na-HEPES, 1.2 $MgSO_4$, 1 KH_2PO_4, 2.5 $CaCl_2$ and 11 glucoses, supplemented with diethyldithiocarbamic acid (1 mmol/L; Sigma-Aldrich) and β-NADPH (0.1 mmol/L; Sigma-Aldrich) at 37ºC for 45 min. The aortas were then transferred to vials containing lucigenin (10 μmol/L; Sigma-Aldrich) in Krebs-HEPES solution. Repeated measurements were recorded by a GloMax® 20/20 Luminometer (Madison, WI, USA) in 1 min intervals for 10 min. The amount of superoxide anion produced was presented as real numbers in relatively light units per mg of dry tissue.

Nitrite assay
Mouse aortas were pretreated with Ach (10 μmol/L) at 37ºC for 10 min to stimulate NO production, followed by nitrate reductase incubation to reduce nitrate to nitrite. Aortas were subsequently homogenized, and supernatants were collected for nitrite level measurement by using a colorimetric assay kit involving the Griess reaction (Molecular Probes, Eugene, USA). Absorbance was recorded at 548 nm and was compared to a standard nitrite curve. Protein contents of homogenates were measured by the Bradford method for nitrite value normalization.

ELISA on circulating inflammatory cytokines
Mouse blood was collected via celiac vein. Serum was obtained by centrifugation at 3000 rpm at room temperature for 10 min. Levels of inflammatory cytokines, including TNFα and IL-6, in mouse serum were measured by ELISA kits (Invitrogen) following the manufacturer's protocol.

Lipid profile
Serum lipid profile was measured by a commercially available assay kit (Stanbio, Boerne, USA) specialized for total cholesterol (TC), triglycerides (TG) and high-density lipoprotein (HDL) cholesterol in serum. HDL was precipitated from the whole serum by adding HDL precipitating reagent (1:10; Stanbio) to serum, followed by a 10-min centrifugation at 1000 xg. Lipid profile was studied following the manufacturer's protocol, where lipid levels were determined by a plate reader (Bio-Rad) at 500 nm. Levels of non-HDL cholesterol were calculated by the formula: non-HDL cholesterol = TC-(TG/5)-HDL.

Endotoxemia detection and intestinal barrier assessment

Translocation of bacterial LPS to circulation and LPS-binding protein (LBP) level were evaluated to detect endotoxemia. Mouse serum endotoxin was measured by the LAL Chromogenic Endotoxin Quantitation kit (Pierce, Massachusetts, USA).

© 2024. Cheng CK et al. Published online at http://www.aginganddisease.org/EN/10.14336/AD.2024.0384

SUPPLEMENTARY DATA

Mouse serum was diluted 1:50-1:100 in pyrogen-free conditions and was inactivated at 70ºC for 15 min. Endotoxin in mouse fecal samples were measured by the Chromogenic kit stated above with a modified protocol. Briefly, fecal pellets were placed in 10 mL PBS in a pyrogen-free tube, followed by a 1-hr sonication. The fecal samples were subsequently centrifugated at 400 xg for 15 min and then filtrated through a 0.22 µm filter. The filtrated samples were subjected to a 1:1000 dilution in pyrogen-free water, followed by a 15-min inactivation at 70ºC. Samples were assayed according to standard kit protocol. Besides, serum levels of LBP and intestinal fatty acid binding protein (I-FABP) were measured by ELISA kits from Invitrogen and MyBioSource (San Diego, USA) respectively, following the manufacturer's protocol.

Statistical analysis

All data were presented as mean ± SD. Statistical analysis was performed by using GraphPad Prism software (Version 8.0). D'Agostino-Pearson test was conducted for normality test. Statistical significance was evaluated by unpaired t-test and nonparametric Mann-Whitney test for two-tailed comparison between two groups. A P value < 0.5 indicated statistical significance.

SUPPLEMENTARY DATA

PART II: SUPPLEMENTARY FIGURES

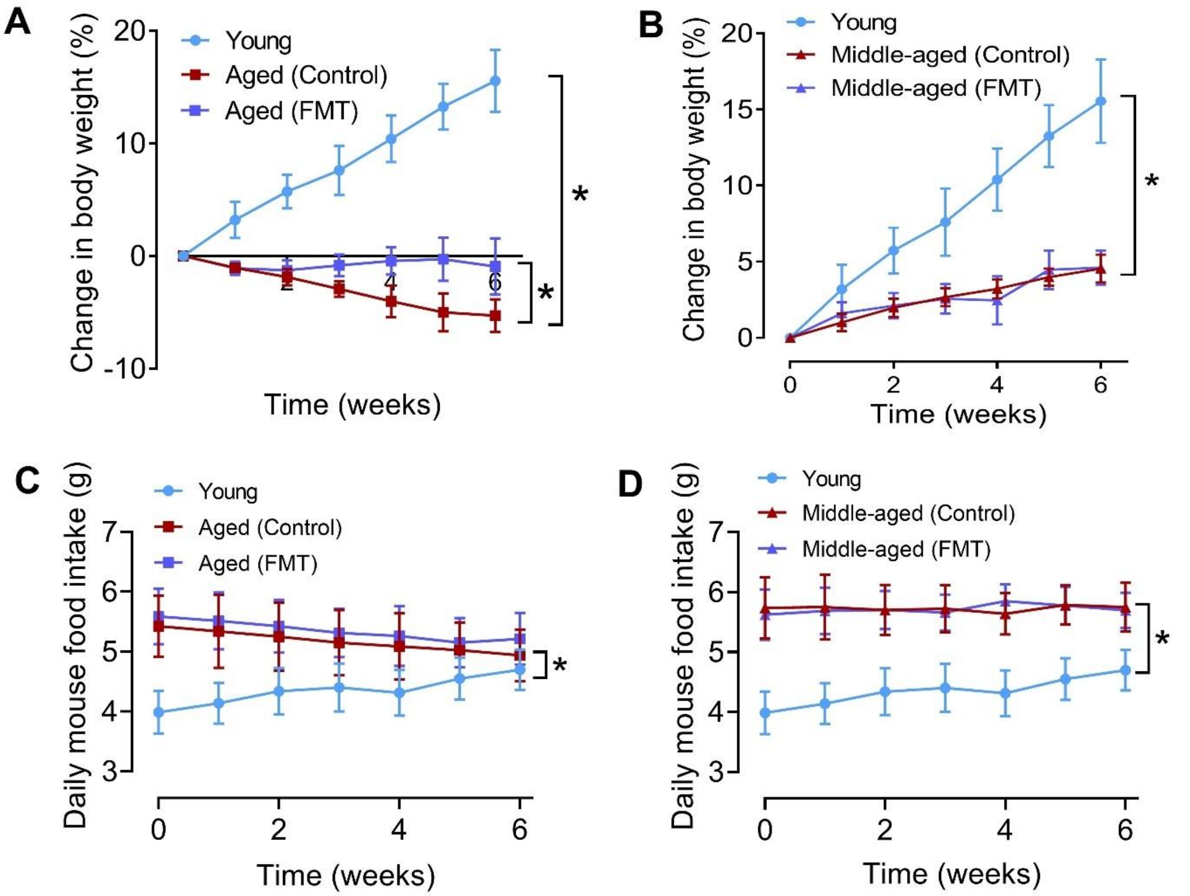

Supplementary Figure 1. Effects of FMT from young donor mice on body weights and food intake of recipient mice. Percentage change in body weights of **(A)** young and aged mice, and **(B)** young and middle-aged mice during the 6-week FMT (n=8 per group). Changes in daily food intake of **(C)** young and aged mice, and **(D)** young and middle-aged mice during the 6-week FMT (n=8 per group). The same curves on body weight change percentage for Young group in **(A)** and **(B)**. The same curves on food intake amount for Young group in **(C)** and **(D)**. Data are mean ± SD. **p*<0.05 (D'Agostino-Pearson normality test, followed by unpaired t-test and nonparametric Mann-Whitney test). FMT, fecal microbiota transfer.

© 2024. Cheng CK et al. Published online at http://www.aginganddisease.org/EN/10.14336/AD.2024.0384

SUPPLEMENTARY DATA

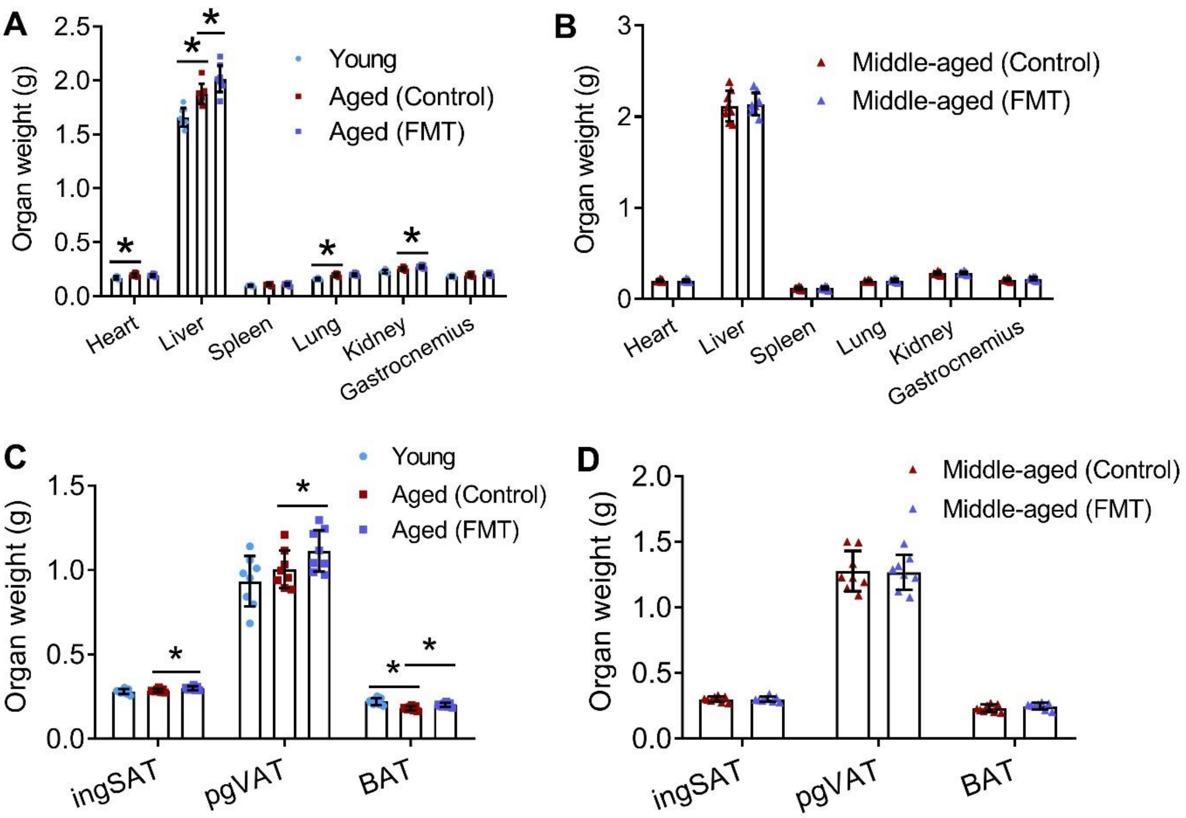

Supplementary Figure 2. Effects of FMT from young donor mice on organ and adipose tissue weights of recipient mice. Weights of indicated organs of **(A)** young and aged mice, and **(B)** middle-aged mice postmortem after the 6-week FMT (n=8 per group). Weights of ingSAT, pgVAT and BAT of **(C)** young and aged mice, and **(D)** middle-aged mice (n=8 per group). Data are mean ± SD. **p*<0.05 (D'Agostino-Pearson normality test, followed by unpaired t-test and nonparametric Mann-Whitney test). BAT, brown adipose tissue; FMT, fecal microbiota transfer; ingSAT, inguinal subcutaneous adipose tissue; pgVAT, perigonadal visceral adipose tissue.

© 2024. Cheng CK et al. Published online at http://www.aginganddisease.org/EN/10.14336/AD.2024.0384

SUPPLEMENTARY DATA

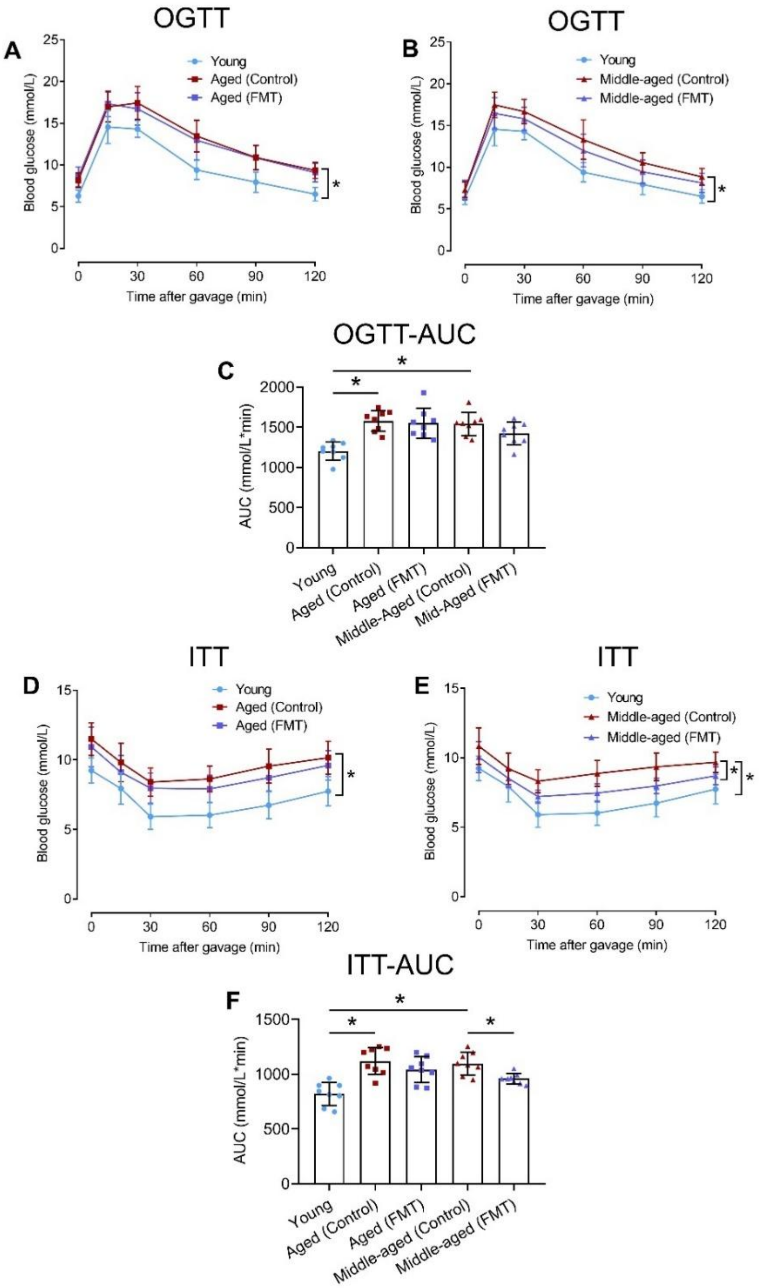

Supplementary Figure 3. Effects of 3-week FMT on glucose homeostasis of aged and middle-aged mice. OGTT of **(A)** young and aged mice, and **(B)** young and middle-aged mice at week 3 of FMT (n=8 per group). **(C)** AUC analysis on OGTT of glucose over time of young, aged, and middle-aged mice at week 3 of FMT (n=8 per group). ITT of **(D)** young and aged mice, and **(E)** young and middle-aged mice at week 3 of FMT (n=8 per group). **(F)** AUC analysis on ITT of glucose over time of young, aged, and middle-aged mice at week 3 of FMT (n=8 per group). The same curves on OGTT results for Young group in **(A)** and **(B)**. The same curves on ITT results for Young group in **(D)** and **(E)**. Data are mean ± SD. $*p<0.05$ (D'Agostino-Pearson normality test, followed by unpaired t-test and nonparametric Mann-Whitney test). AUC, area under curve; FMT, fecal microbiota transfer; ITT, insulin tolerance test; OGTT, oral glucose tolerance test.

© 2024. Cheng CK et al. Published online at http://www.aginganddisease.org/EN/10.14336/AD.2024.0384

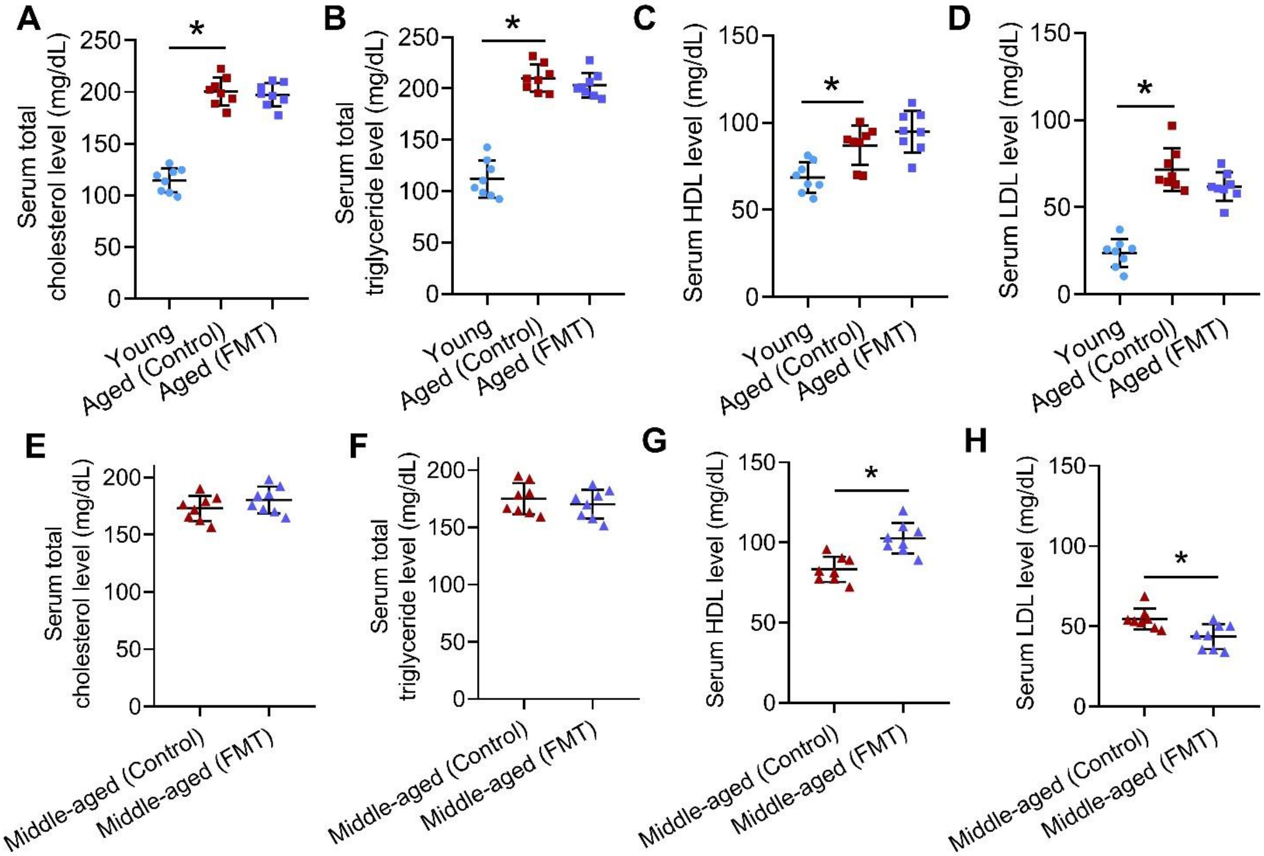

Supplementary Figure 4. Lipid profiles of aged and middle-aged mice after the 6-week FMT. Serum levels of **(A)** total cholesterol, **(B)** total triglycerides, **(C)** HDL cholesterol, and **(D)** LDL cholesterol of young and aged mice (n=8 per group). Serum levels of **(E)** total cholesterol, **(F)** total triglycerides, **(G)** HDL cholesterol, and **(H)** LDL cholesterol of middle-aged mice (n=8 per group). Data are mean ± SD. *p<0.05 (D'Agostino-Pearson normality test, followed by unpaired t-test and nonparametric Mann-Whitney test). FMT, fecal microbiota transfer; HDL, high-density lipoprotein; LDL, low-density lipoprotein**.**

© 2024. Cheng CK et al. Published online at http://www.aginganddisease.org/EN/10.14336/AD.2024.0384

SUPPLEMENTARY DATA

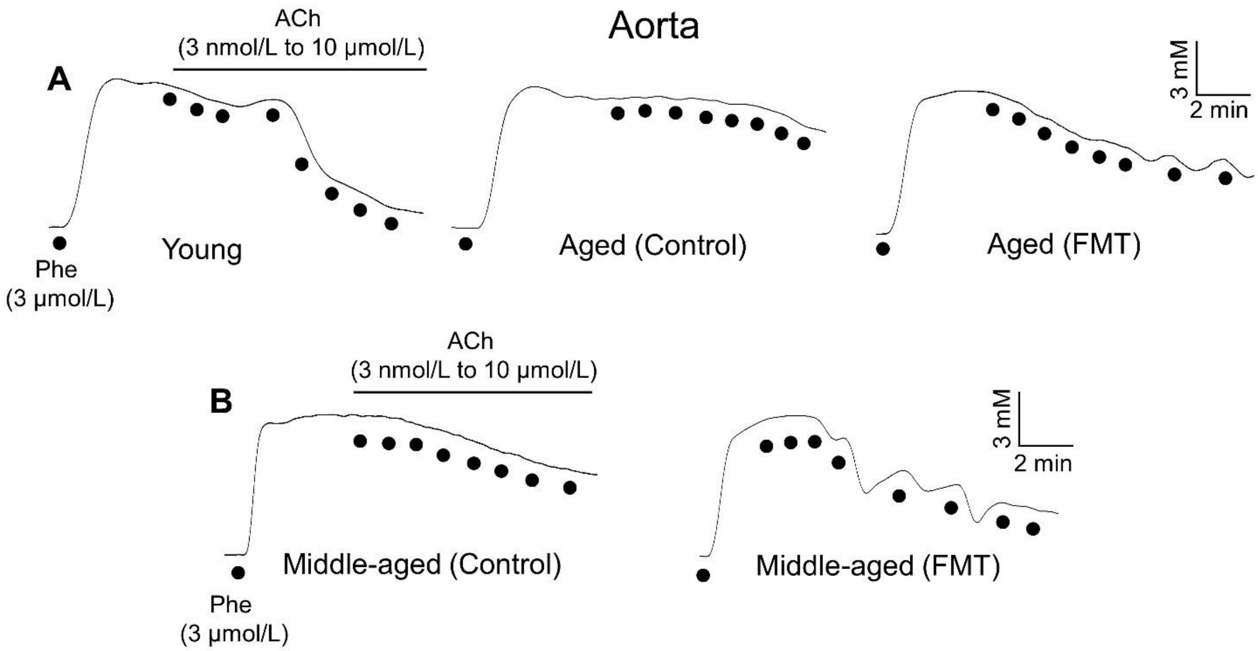

Supplementary Figure 5. Representative trace of wire myography on aortas of aged and middle-aged mice. Representative traces for EDRs in aortas of **(A)** young and aged mice, and **(B)** middle-aged mice. ACh, acetylcholine; EDR, endothelium-dependent relaxation; FMT, fecal microbiota transfer; Phe, phenylephrine.

© 2024. Cheng CK et al. Published online at http://www.aginganddisease.org/EN/10.14336/AD.2024.0384

SUPPLEMENTARY DATA

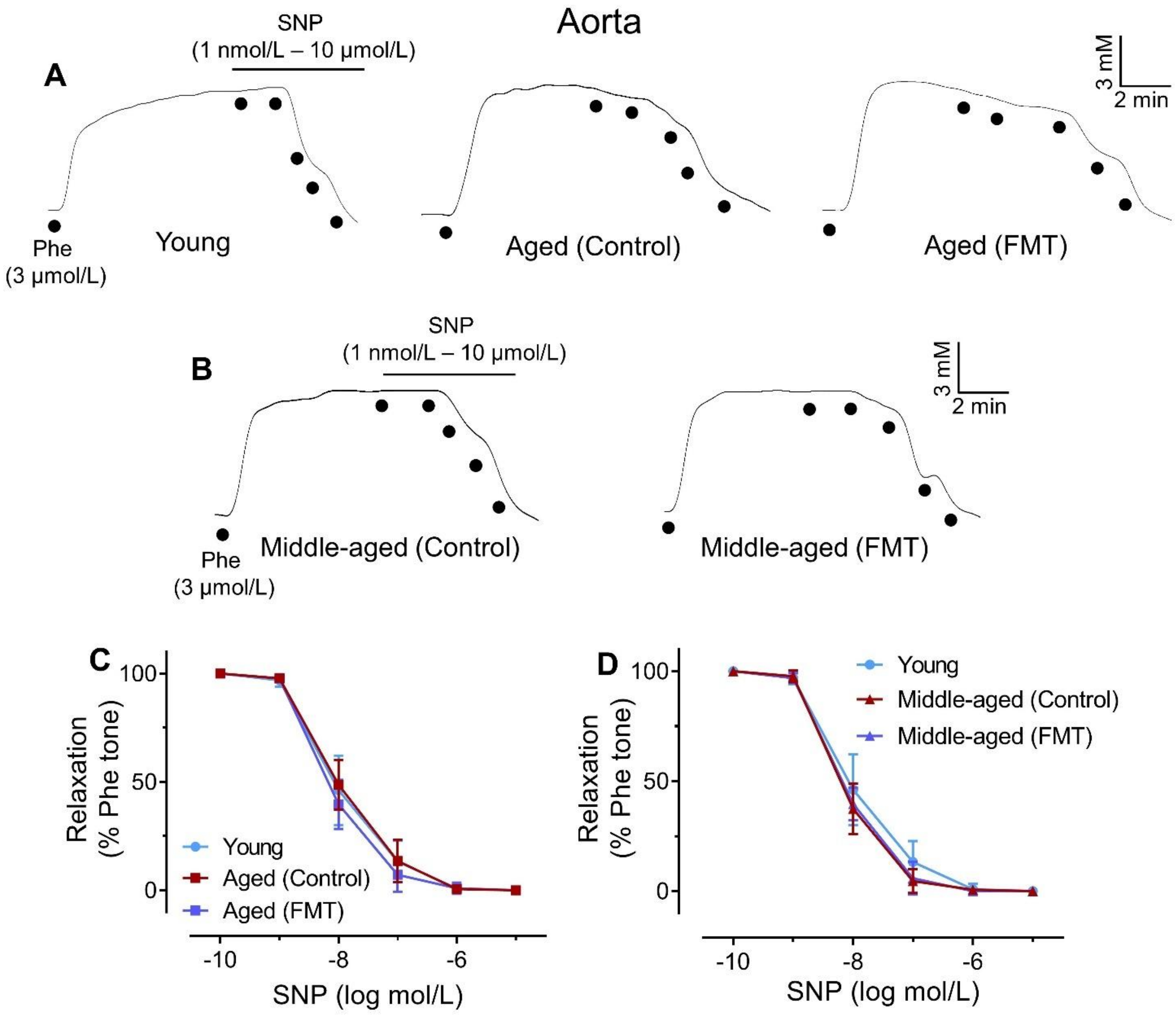

Supplementary Figure 6. SNP-induced endothelium-independent vasodilation on aortas of aged and middle-aged mice. Representative traces for SNP-induced endothelium-independent relaxations in aortas of **(A)** young and aged mice, and **(B)** middle-aged mice. Summary statistics of wire myography on endothelium-independent relaxations in aortas of **(C)** young and aged mice, and **(D)** young and middle-aged mice (n=8 per group). The same curves on SNP-induced vasorelaxation for Young group in **(C)** and **(D)**. Data are mean ± SD. (D'Agostino-Pearson normality test, followed by unpaired t-test and nonparametric Mann-Whitney test). FMT, fecal microbiota transfer; Phe, phenylephrine; SNP, sodium nitroprusside.

© 2024. Cheng CK et al. Published online at http://www.aginganddisease.org/EN/10.14336/AD.2024.0384

SUPPLEMENTARY DATA

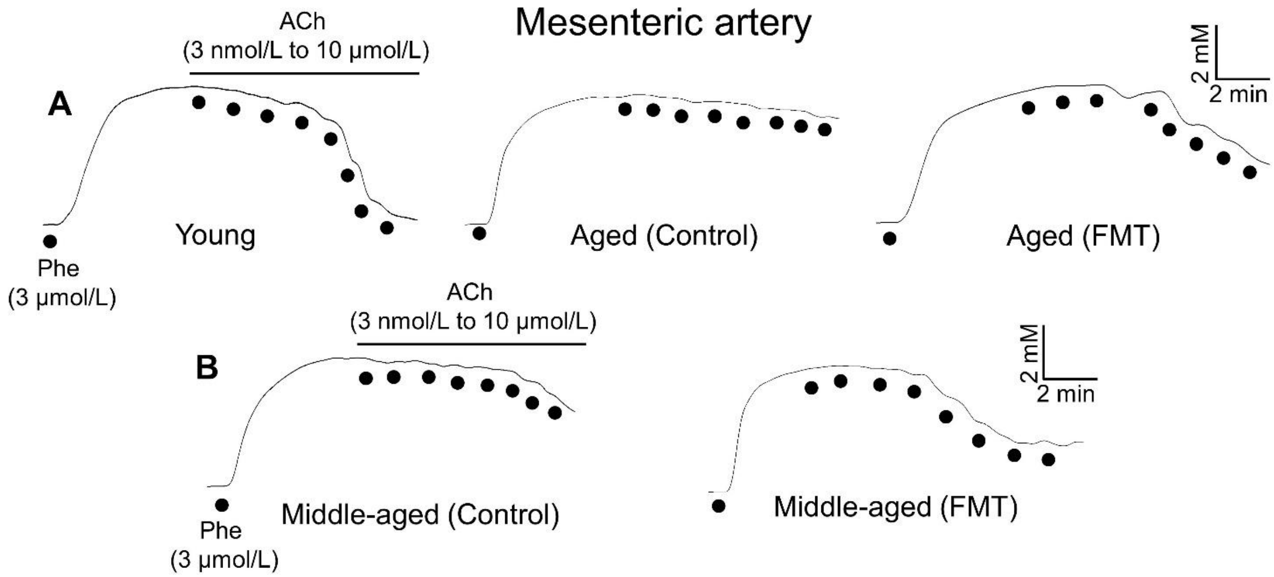

Supplementary Figure 7. Representative trace of wire myography on mesenteric arteries of aged and middle-aged mice. Representative traces for EDRs in aortas of **(A)** young and aged mice, and **(B)** middle-aged mice. ACh, acetylcholine; EDR, endothelium-dependent relaxation; FMT, fecal microbiota transfer; Phe, phenylephrine.

© 2024. Cheng CK et al. Published online at http://www.aginganddisease.org/EN/10.14336/AD.2024.0384

SUPPLEMENTARY DATA

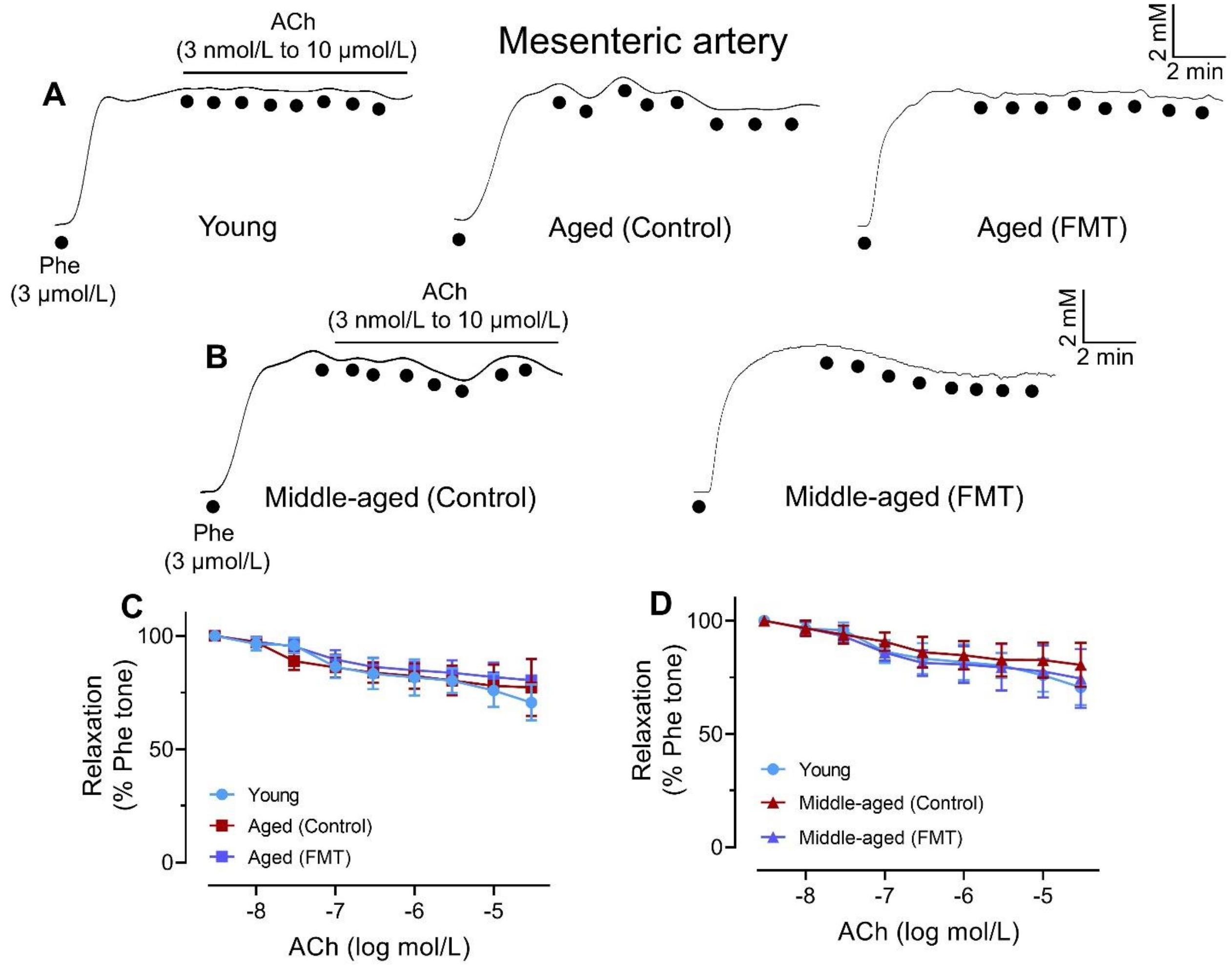

Supplementary Figure 8. Representative traces and summarizing results of wire myography on mesenteric arteries of aged and middle-aged mice upon L-NAME incubation. Representative traces for EDRs in mesenteric arteries of **(A)** young and aged mice, and **(B)** middle-aged mice upon NG-nitro-L-arginine methyl ester (L-NAME) incubation. Summary statistics of wire myography on EDRs in mesenteric arteries of **(C)** young and aged mice, and **(D)** young and middle-aged mice upon L-NAME incubation (n=8 per group). The same curves on ACh-induced vasorelaxation for Young group in **(C)** and **(D)**. Data are mean ± SD. (D'Agostino-Pearson normality test, followed by unpaired t-test and nonparametric Mann-Whitney test). ACh, acetylcholine; EDR, endothelium-dependent relaxation; FMT, fecal microbiota transfer; L-NAME, NG-nitro-L-arginine methyl ester; Phe, phenylephrine.

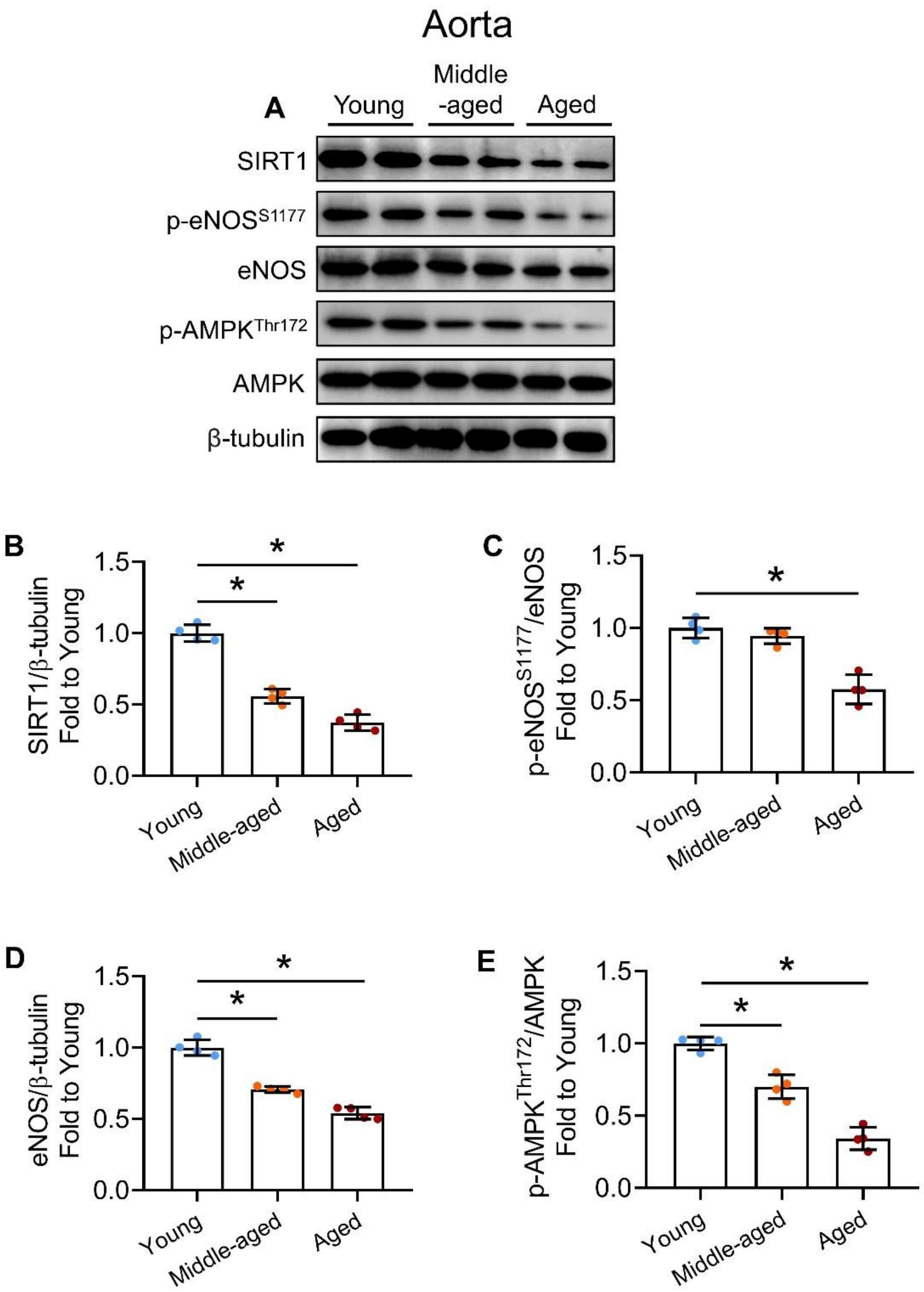

Supplementary Figure 9. Effects of aging on vascular function-related signaling in aortas. **(A)** Representative Western blots on expression of AMPK, p-AMPK at Thr172, eNOS, p-eNOS at Ser1177 and SIRT1 in aortas of young, middle-aged and aged mice. Quantification of Western blotting on expression of **(B)** SIRT1, **(C)** p-eNOS at Ser1177, **(D)** eNOS, and **(E)** p-AMPK at Thr172 in aortas of young, middle-aged and aged mice (n=4 per group). Data are mean ± SD. **p*<0.05 (D'Agostino-Pearson normality test, followed by unpaired t-test and nonparametric Mann-Whitney test).

© 2024. Cheng CK et al. Published online at http://www.aginganddisease.org/EN/10.14336/AD.2024.0384

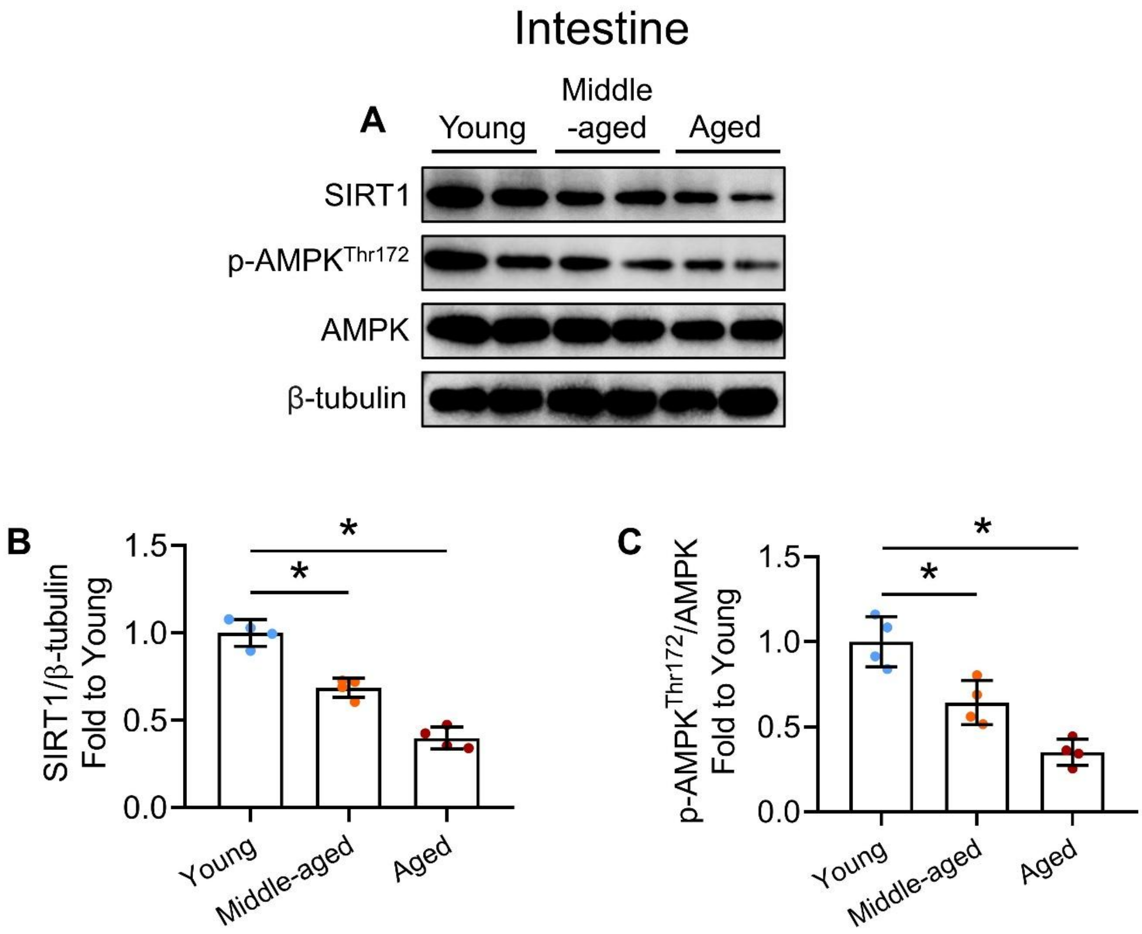

Supplementary Figure 10. Effects of aging on AMPK/SIRT1 signaling axis in intestines. (A) Representative Western blots on expression of AMPK, p-AMPK at Thr172 and SIRT1 in intestines of young, middle-aged, and aged mice. Quantification of Western blotting on expression of **(B)** SIRT1, and **(C)** p-AMPK at Thr172 in aortas of young, middle-aged and aged mice (n=4 per group). Data are mean ± SD. *p<0.05 (D'Agostino-Pearson normality test, followed by unpaired t-test and nonparametric Mann-Whitney test).

© 2024. Cheng CK et al. Published online at http://www.aginganddisease.org/EN/10.14336/AD.2024.0384

SUPPLEMENTARY DATA

Part III: Supplementary Table

Supplementary Table 1. Primer sequences for quantitative RT-PCR.

Genes	Forward (5′-3′)	Reverse (5′-3′)
E-selectin	AGTTGTGAGTTCTCCTGCGA	CACTCCATGACGCCATTCTG
Icam1	GTGATGCTCAGGTATCCATCCA	CACAGTTCTCAAAGCACAGCG
Il-6	TTCAGCCCTTGCTTGCCTC	ACACTTTTACTCCGAAGTCGGT
Tnfα	CAGCCTCTTCTCATTCCTGC	ATGAGAGGGAGGCCATTTG
Vcam1	GTTCCAGCGAGGGTCTACC	AACTCTTGGCAAACATTAGGTGT
Tert	CAGCCATACATGGGCCAGTTC	ACAGGCTGCTGCTGCTCTCA
Gapdh	AGGTCGGTGTGAACGGATTTG	TGTAGACCATGTAGTTGAGGTCA
Telomere	CGGTTTGTTTGGGTTTGGGTT	GGCTTGCCTTACCCTTACCCT
	TGGGTTTGGGTTTGGGTT	TACCCTTACCCTTACCCT
36B4	ACTGGTCTAGGACCCGAGAAG	TCAATGGTGCCTCTGGAGATT

© 2024. Cheng CK et al. Published online at http://www.aginganddisease.org/EN/10.14336/AD.2024.0384
